# Bidirectional Trust-Enhanced Collaborative Filtering for Point-of-Interest Recommendation

**DOI:** 10.3390/s23084140

**Published:** 2023-04-20

**Authors:** Jingmin An, Wei Jiang, Guanyu Li

**Affiliations:** College of Information Science and Technology, Dalian Maritime University, Dalian 116026, China; anjingmin@dlmu.edu.cn (J.A.); merryaha@dlmu.edu.cn (W.J.)

**Keywords:** POI recommendation, bidirectional trust, collaborative filtering, weighted matrix factorization, data sparsity

## Abstract

A personalized point-of-interest (POI) recommender system is of great significance to facilitate the daily life of users. However, it suffers from some challenges, such as trustworthiness and data sparsity problems. Existing models only consider the trust user influence and ignore the role of the trust location. Furthermore, they fail to refine the influence of context factors and fusion between the user preference and context models. To address the trustworthiness problem, we propose a novel bidirectional trust-enhanced collaborative filtering model, which investigates the trust filtering from the views of users and locations. To tackle the data sparsity problem, we introduce temporal factor into the trust filtering of users as well as geographical and textual content factors into the trust filtering of locations. To further alleviate the sparsity of user-POI rating matrices, we employ a weighted matrix factorization fused with the POI category factor to learn the user preference. To integrate the trust filtering models and the user preference model, we develop a fused framework with two kinds of integrating methods in relation to the different impacts of factors on the POIs that users have visited and the POIs that users have not visited. Finally, we conduct extensive experiments on Gowalla and Foursquare datasets to evaluate our proposed POI recommendation model, and the results show that our proposed model improves by 13.87% at precision@5 and 10.36% at recall@5 over the state-of-the-art model, which demonstrates that our proposed model outperforms the state-of-the-art method.

## 1. Introduction

Currently, the rapid development of the social internet of things is promoting the realization of a smart city [1]. With the growing popularization of modern mobile communication technologies (i.e., mobile devices, wireless networks, and 5G communication), more and more location-based social networks (LBSNs) have emerged, such as Gowalla, Foursquare, and Weibo, etc., where users are able to instantaneously share locations visited by them. In relation to this, if we can obtain and make full use of the collected information, including user check-in records and contexts from LBSNs, the accuracy of personalized POIs recommended to users would then greatly improve. Furthermore, with a user’s POI information, it is possible to know when the user is at which location and how often he/she appears at the location. This will not only help us to explore potential customers but also design advertisements to attract customers.

In LBSNs, a personalized POI recommendation aims to recommend unvisited POIs to targeted users in terms of users’ historical check-ins [2,3], which is of great significance in the realization of a smart city. This is because the received recommendation results are beneficial for users’ personalized demands, users’ behavior trends or trajectory predictions, as well as certain traffic fields related to users’ behavior trends. However, to accurately predict whether a personalized user will visit a specific location at a specific time based on historical check-ins, it is a critical challenge to cope with the issue of data sparsity and trustworthiness. For example, in real-world Gowalla and Foursquare datasets, the user-POI checking-in density is 0.291% and 0.385%, respectively, and the user-POI rating matrix contains some noise [4,5], i.e., fake ratings and malicious feedbacks.

For this study, we have divided existing works into two categories:

(1) **Fused multiple factors models** [6,7,8,9,10,11]: These works consider the roles of various context factors in order to determine and match user preferences, i.e., geographical, social, temporal, POI category, and textual content factors. The authors employ different advanced techniques to integrate these factors for POI recommendations, i.e., matrix factorization, convolution neural network, graph neural network, recurrent neural network, and deep neural network. However, all of them assume the user-POI rating is reliable and ignore the unreliable rating involved in check-ins. There is no doubt that if unreliable noise data are not excluded, the accuracy of recommendation is limited regardless of the model’s optimization.
(2) **Trust-enhanced models** [12,13,14,15]: These works [12,13,14] propose a user–user trust matrix modeled by calculating the trust relationship between users. Combining the user trust matrix and the user rating, they achieve the trust-enhanced similarity between users. The work [15] proposes a DeepWalk-based trust similarity measurement running over a co-visited network. Nevertheless, all of them ignore the temporal factor influence on the user similarity, leading to biased similarities. For example, the user $u_{k}$ visits POI $l_{1}$ and $l_{2}$ at time $t_{1}$ and $t_{2}$, respectively, while $u_{i}$ checks in $l_{2}$ and $l_{1}$ at time $t_{1}$ and $t_{2}$, respectively. When not considering time, the similarity between the two users is 1. However, if taking time into account, the similarity between them is 0, since the time of their check-ins on the two POIs is different. Existing methods fail to distinguish the difference. Furthermore, these authors only consider the trust filtering from the view of users, resulting in insufficient trustworthiness evaluations. For example, given a location $l_{j}$ and the check-in set $L_{i}\left(l_{j}\notin L_{i}\right)$ visited by $u_{i}$, $l_{j}$ could be recommended to $u_{i}$ due to their stronger correlations on geographical or textual content factors. However, $l_{j}$ may not be a good choice for $u_{i}$ when it is rated by other unreliable users. Existing methods fail to consider the trust filtering from the view of locations.

To summarize, existing works suffer from two shortcomings:

- These works fail to refine the influence of context factors and fusion between the user preference and context models;
- These works ignore the role of the trust filtering from the view of locations.

To make up the shortcomings of existing works, we are the first to propose a novel bidirectional trust-enhanced collaborative filtering model for both trustworthiness and data sparsity problems. We have developed a trustworthy community combining the direct trust and the indirect trust between users, where the collaborative filtering is performed from the views of users and locations via leveraging temporal, geographical, and textual content factors. Secondly, to further alleviate the sparsity of user-POI rating matrices, we integrate a POI category factor into a weighted matrix factorization to learn the user preference. Thirdly, we develop a fused framework with two kinds of integrating methods for the trust filtering and the user preference models, which considers the different impacts of factors on the POIs that users have visited and the POIs that users have not visited. Finally, we conduct extensive experiments on two real-world datasets to evaluate our proposed POI recommendation model and baseline methods.

To sum up, our main contributions are as follows:

- We propose a novel bidirectional trust-enhanced collaborative filtering model, which performs the trust filtering from the views of users and locations via leveraging temporal, geographical, and textual content factors. To our knowledge, we are the first to focus on trust filtering from the views of users and locations.
- We refine the influence of context factors against the data sparsity problem.
- We develop a fused framework for the trust filtering and the user preference models, which considers the different impacts of factors on the POIs that users have visited and the POIs that users have not visited.

The remainder of the paper is organized as follows. In Section 2, we review some state-of-the-art-related works. We present the overview and technical details of our proposed recommendation model in Section 3. In Section 4, we present our experimental setup and results. In Section 5, we conclude our work.

## 2. Related Works

In this paper, our proposed model aims to address the data sparsity and trustworthiness problems in POI recommender systems, and thus we review the related literature, including fused multiple context factors POI recommendations against data sparsity problem and trust-enhanced POI recommendations filtering out noise information.

### 2.1. Fused Multiple Factors POI Recommendations

To alleviate data sparsity, more and more work focus on exploring the roles of different context factors, i.e., geographical, social, temporal, POI category, and textual content factors. Gao et al. [16] model four types of friendships based on the geographical location and social relationship. Li et al. [17] propose three types of friendships, including social friends, location friends, and neighboring friends, which are fused into a unified matrix factorization framework with two loss functions for POI recommendation. Since user check-in behaviors vary with time, capturing user check-in temporal features is essential. Yuan et al. [18] propose a temporal factor-enhanced collaborative filtering model to recommend POIs at a specific time. Gao et al. [19] propose a spatiotemporal recommender network model to recommend unvisited POIs to a given user at a specific time in a day. However, these methods only consider a few context factors such as geographical, social, and temporal factors, causing a limited alleviation of data sparsity. Compared with them, our proposed model considers more context factors in predicting user POIs.

Furthermore, to utilize more context factors, some hybrid models have been proposed. Zhang et al. [6] propose a fused matrix factorization with multi-tag, social, and geographical factors for POI recommendation, where the multi-tag factor is extracted from the user-POI rating matrix. Xing et al. [7] propose a content-aware POI recommendation based on the convolutional neural network, which introduces three types of content information, including POI properties, user interests, and sentiment indications. Davtalab et al. [8] propose a probabilistic matrix factorization model integrating social spatiotemporal information, which develops a multivariable inference approach using the latent social space, geographical space, and POI category space similarities for POI recommendation. Liu et al. [9] argue that spatial and temporal effects should be analyzed simultaneously in POI recommendation, and they design a four-way neural interaction model over a spatiotemporal heterogeneous information network to mine user preferences. In addition, some works integrate various contexts to capture the user’s next POIs in terms of his/her current check-in trajectory. Li et al. [10] propose an attention-based spatiotemporal gated graph neural network model for sequential POI recommendations. Dai et al. [20] propose a unified spatiotemporal neural network framework via leveraging users’ check-in records and social ties, and the framework recommends POIs to users by joint embedding and sequential modeling. Chakraborty et al. [21] propose an unsupervised, generic framework involving a factored relevance model, which balances this trade-off between user historical preferences and current preferences. Yu et al. [22] propose a top-*K* initial POI recommendation model by considering the influence of similarity, popularity, and location of POIs, which is then used to further achieve subsequent recommendations through transfer probability. Wu et al. [11] present a personalized next POI recommendation framework, where they employ a long- and short-term method to learn the specific preference for each user by fusing the POI category and check-in time information. Xu et al. [23] develop a multi-modal collaborative filtering which focuses on addressing efficient cold-start recommendation. They map multi-modal auxiliary features of users and items into binary hash codes and leverage a novel discrete optimization strategy to learn the user and item codes, reducing the storage cost in the hash optimization process. Considering that existing recommender systems fail to explain recommendations to users, Zhu et al. [24] propose an explainable discrete collaborative filtering, which is a multi-task learning framework fully exploiting the correlations between the preference prediction task and the explanation generation task based on hash codes for users and items. Margaris et al. [25] propose a novel collaborative filtering with the experiencing period criterion, aiming to keep recommendations close to users’ patterns of practice. It can delay recommending a newly released item to users that prefer to delay the experience of new items in a particular category, while recommending these new items to other users who intend to experience trendy items in time. Wang et al. [26] develop a novel spatial-temporal and text representation learning framework which can learn long-term dependencies among visits in check-in sequences, users’ perspectives, and POIs’ reputations from textual reviews. Fang et al. [27] construct the user–POI interaction graph, the user social graph, and the POI geographical vector, which are fused via a graph neural network for POI recommendations. Nevertheless, these methods ignore the unreliable rating involved in check-ins, which lead to limited accuracy, although they employ different techniques to optimize the recommendation model. In contrast, our proposed model considers trust filtering from the views of users and locations.

### 2.2. Trust-Enhanced POI Recommendations

To filter out noise information in user rating matrices and achieve trust-enhanced recommendations, some works take the trust relationship between users in LBSNs into account. Deng et al. [28] propose a relevant trust walker to generate personalized POI recommendations, which enhances the trust relationship between similar users. Logesh et al. [12] propose a social pertinent trust walker model, which utilizes a modified random walk based on trust pertinence calculated by matrix factorization for POI recommendation. Liu et al. [29] propose a trust-aware recommendation model, where the trust relationship is measured by combining explicit and implicit trust between users. Ahmadian et al. [30] propose an effective neighbor selection mechanism for removing unreliable users from the nearest neighbor set, which enhance the accuracy of recommendations. Guo et al. [31] leverage implicit feedbacks of users to measure trust relationships between users. The trust relationship is used to model three factored similarities. Guo et al. [32] propose three heterogeneous graphs, including user–item interaction graph, user–user trust relation graph, and item–item knowledge graph. They present recommendations to users by fusing these heterogeneous graphs. Ahmed et al. [33] and Ma et al. [34] integrate the user trust relationship into deep neural networks for cross-domain recommendations, respectively. Zhao et al. [35] propose a fused user trust relationships tensor factorization model to address trustworthiness and data sparsity problems, where the trust relationships are divided into unilateral trust and mutual trust for an improved use of social information. Wang et al. [15] propose a trust-enhanced collaborative filtering model which fuses the geographical influence, temporal influence, and trust relationship learned by DeepWalk model running over the user co-visiting network. Ahmadian et al. [13,14] develop a trust matrix from user trust networks that are modeled by combining the similarities of user implicit ratings and the trust relationship between users. Additionally, the authors input trust matrices, user-item rating matrices, and user-tag matrices into a deep neural network to learn user preferences. These methods employ different techniques such as random walk, deep walk, and trust matrix to enhance trustworthiness. However, they only consider trust filtering from the view of users, resulting in insufficient trustworthiness evaluations. In contrast, our proposed model performs trust enhancement from the views of users and locations.

## 3. Proposed Recommendation Model

### 3.1. Problem Formulation

**Figure 1:**
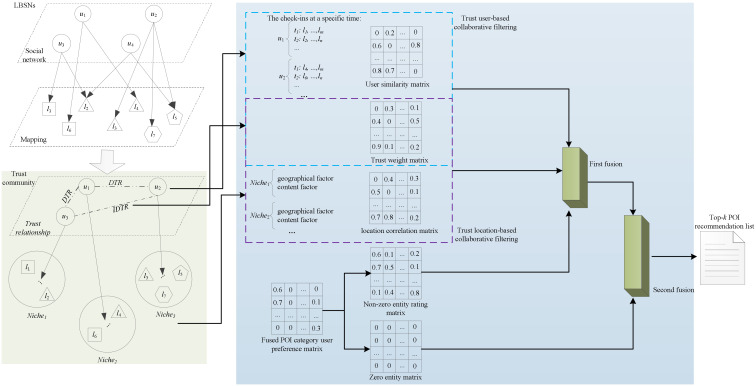
The overview of the proposed model. Green-filled area represents the proposed trust community framework, and the blue one denotes the methodology based on the trust community framework, including the trust-enhanced collaborative filtering from the views of users and locations and the two-step data fusion.

We focus on the research of a novel POI recommendation model with bidirectional trust filtering, which recommends unknown or unvisited POIs to targeted users in LBSNs on the premise of reliable users and locations, as illustrated in Figure 1. Firstly, to establish the trust weighted matrix, we propose a trustworthy community modeled by the combination of direct and indirect trusts, where reliable users and their corresponding visited POIs are included. We perform the collaborative filtering from the views of these users and locations in the trustworthy community. From the point of view of users, we propose a time-aware similarity measurement between users for modeling the user similarity matrix. We fuse the trust-weighted matrix and the user-similarity matrix to perform the trust user-based collaborative filtering and capture the trust user influence matrix. From the point of view of locations, we develop a location correlation measurement by leveraging geographical and content factors for modeling the POI geographical correlation matrix. We fuse the trust-weighted matrix and the geographical correlation matrix to achieve the trust location-based collaborative filtering and obtain the trust location influence matrix. Secondly, we integrate the POI category factor into a weighted matrix factorization to learn the user preference matrix to address sparse user-POI rating matrices. Thirdly, we present two kinds of integrating methods for the trust filtering and the user preference models, and the first fusion is to integrate the non-zero entities in the user preference matrix into the trust filtering models, and the second fusion is to take zero entities in the user preference matrix into the first fusion. Afterward, we can achieve top-*k* POIs that the target user would be interested in. The descriptions of some key notions are presented in Table 1.

**Definition** **1.** *(point-of-interest, POI*$l_{j}$*). Let* $l_{j}=\left(l_{j}.loc,\;l_{j}.doc\right)$*, where* $l_{j}.loc$*represents the geographical location of* $l_{j}$*, and* $l_{j}.doc$ *indicates the textual content of* $l_{j}$*. Let a POI set be* $L=\left\{l_{1},l_{2},\cdots ,l_{j}\right\}$.

**Definition** **2.** *(trustworthy community, TC). Let* TC=(U,Nic)*,* $U=\left\{u_{1},u_{2},\cdots ,u_{k}\right\}$ *is a trust user set of* $u_{i}$*, where there are the trust relationships between* $u_{k}$ *and* $u_{i}$. $Nic=\left\{Niche_{1},Niche_{2},\cdots ,\;Niche_{k}\right\}$*, where* $Niche_{k}$ *is a set of locations that are visited by* $u_{k}$.

**Definition** **3.** *(trust relationship, TR). TR consists of direct trust (DTR) and indirect trust (IDTR). DTR: if* $u_{k}$ *and* $u_{i}$ *covisit POIs* L*, then there is DTR between* $u_{k}$ *and* $u_{i}$*. IDTR:* $u_{k}$ *and* $u_{i}$ *do not covisit POIs* L*, but if there is DTR between* $u_{k}$ *and* $u_{p}$ *as well as* $u_{p}$ *and* $u_{i}$*, then there is IDTR between* $u_{k}$ *and* $u_{i}$.

### 3.2. Modeling Trustworthy Community from LBSNs

We believe that it is an effective method to obtain *DTR* whereby both recommended users and the target user directly participate in the interactions of check-in POIs, where the trust is measured according to their ratings. Due to the applicability of the beta trust model [36] in building trust relationships based on a large number of ratings, we employ it to measure *DTR* between users. Moreover, compared with other clustering- and graph-learning methods, the beta model is simpler, more practical, and has lower time complexity. Let both $u_{k}$ and $u_{i}$ check in at $l_{j}$, and the ratings of $u_{k}$ and $u_{i}$ on $l_{j}$ are $r_{k,j}$ and $r_{i,j}$, respectively. When $\left|r_{k,j}-r_{i,j}\right|$ is less than a fixed value, the recommendation to $u_{i}$ leveraging $u_{k}$’s preference is positive, when otherwise negative. $n_{k,i}$ and ${\bar{n}}_{k,i}$ are, respectively, the frequency of positive and negative recommendations. We use the beta probability density function to calculate the posterior probabilities (ρ, 1−ρ) of positive and negative recommendations. $DTR_{k,i}$ can be calculated as follows:

(1) $$DTR_{ki}=\frac{\Gamma ({\bar{n}}_{k,i}+n_{k,i}+2)}{\Gamma ({\bar{n}}_{k,i}+1)\Gamma (n_{k,i}+1)}\rho^{n_{k,i}}(1-\rho {)}^{{\bar{n}}_{k,i}}$$

where Γ is the gamma function, 0≤ρ≤1. In recommender systems, the sparse phenomenon of the user interaction is common, thus a *DTR* between two users may not exist. We believe that *IDTR* mining is very essential to enhance the completeness of the trust relationship. As Definition 3 illustrated, *IDTR* can be measured through the transfer of *DTR*. When *D* is a set consisting of the users that have *DTR* with the target user, the proposed $IDTR_{ki}$ can then be calculated by combining $DTR_{kp}$, as follows:

(2) $$IDTR_{ki}=\frac{\sum_{u_{p}\in D}DTR_{pi}\cdot DTR_{kp}}{\sum_{u_{p}\in D}DTR_{pi}}$$

Based on $DTR_{ki}$ and $IDTR_{ki}$, we can gain the trust relationship between $u_{k}$ and $u_{i}$ by:

(3) $$TR_{ki}=\alpha DTR_{ki}+(1-\alpha )IDTR_{ki}$$

where α is a weighted parameter which denotes the relative importance of *DTR* to *TR*, and 0≤α≤1. We adopt a threshold θ to eliminate unreliable users and reserve trustworthy ones. When $TR_{ki}\geq \theta$, $u_{k}$ and corresponding $Niche_{k}$ are reserved, or otherwise eliminated.

### 3.3. Trust User-Based Collaborative Filtering

We measure the similarities between $u_{k}$ and $u_{i}$ from two aspects. One is the geographical similarity. According to Tobler’s First Law of Geography [37], everything is related to everything else, but near objects are more related with each other than distant objects. Thus, we believe that closer users are more similar. We employ the kernel function triangular method with nonparametric to estimate the user similarity on geography. Triangular can filter out users with low geographical correlations in contrast to other kernel functions (i.e., Gaussian). Specifically, the geographical similarity $gs_{ki}$ can be calculated as follows:

(4) $$gs_{ki}=(1-\frac{d(u_{k},u_{i})}{b})I_{b}$$

where $d(u_{k},u_{i})$ returns the geographical distance between their residences. b is a width of the kernel function. $I_{b}$ is an indicator function (if $d(u_{k},u_{i})\leq b$, then $I_{b}=1$, otherwise $I_{b}=0$). Thus, when $d(u_{k},u_{i})\leq b$, the smaller $d(u_{k},u_{i})$ is, and the larger $gs_{ki}$ is.

Another similarity is checking-in behavior similarity. We argue that the checking-in similarity between users should be considered by the same POIs and the similar POIs visited by them. For instance, $u_{k}$ and $u_{i}$ have visited $l_{j}$, which could show that they have a similar preference. Additionally, $u_{k}$ has visited $l_{j}$ and $u_{i}$ has checked in $l_{p}$, and $l_{j}$ and $l_{p}$ belong to the same category, which could show that they have a similar preference to some extent. Simultaneously, we observe that the checking-in frequency and time are important for the similarity measurement. The higher the frequency of both $u_{k}$ and $u_{i}$ visiting $l_{j}$, the higher the similarity between $u_{k}$ and $u_{i}$. The more consistent the time of both $u_{k}$ and $u_{i}$ checking-in $l_{j}$ is, the higher the similarity between $u_{k}$ and $u_{i}$ is. Therefore, we present a novel similarity measurement by taking these mentioned factors into account. We utilize CBOW model [38] to learn user checking-in behavior patterns and calculate the similarity with Earth Mover’s Distance [39] $EMD\left(u_{k},u_{i}\right)$.

Let the behavior patterns of $u_{i}$ and $u_{k}$ at time t be $W_{i}^{t}$ and $W_{k}^{t}$, respectively.$\;W_{i}^{t}=\left\{\left(w_{p1},\;c_{p1}\right),\left(w_{p2},\;c_{p2}\right)\;\ldots ,\left(w_{pm},\;c_{pm}\;\right)\right\}$ and $W_{k}^{t}=\{\left(w_{q1},\;c_{q1}\right),\left(w_{q2},\;c_{q2}\right),$ $\ldots ,\;\left(w_{qn},\;c_{qn}\;\right)\}$, where $w_{i}$ is a word indicating $l_{j}$, and $c_{i}$ is the normalized frequency of $w_{i}$. T is a piece of a set of time slots, T={1,2,⋯,24}, t ∈T. Note that we integrate temporal factor into user checking-in behavior patterns to distinguish the check-ins at different times, leading to magnifying data sparsity. To address this challenge, we adopt a smoothing check-in method. Let the check-in vector corresponding to $W_{i}^{t}$ be ${\mathbb{C}}_{i,t}=\left\{C_{i,1,t\;},C_{i,2,t},\cdots ,C_{i,j,t}\right\}$, where $C_{i,j,t}=1$ if $u_{i}$ has visited $l_{j}$ at time t, and $C_{i,j,t}=0$, or otherwise. We propose to utilize the similarity $\eta_{t,t^{\prime }}$ between the vectors at t and $t^{\prime }$ measured by Cosine similarity to smooth ${\mathbb{C}}_{i,t}$ as follows:

(5) $${\hat{C}}_{i,j,t}=\overset{T}{\underset{t^{\prime }=1}{\sum }}\frac{\eta_{t,t^{\prime }}}{\sum_{t^{\prime \prime }=1}^{T}\eta_{t,t^{\prime \prime }}}C_{i,j,t^{\prime }}\;$$

and we add ${\hat{C}}_{i,j,t}$ to ${\mathbb{C}}_{i,t}$ for updating $W_{i}^{t}$. Similarly, we update $W_{k}^{t}$ via smoothing ${\mathbb{C}}_{k,t}$. Such improved $EMD\left(u_{k},u_{i}\right)$ can be calculated as follows:

(6) $$EMD\left(u_{k},u_{i}\right)=\underset{f_{ki}}{\min}\frac{\sum_{t=1}^{T}\sum_{i=1}^{m}\sum_{k=1}^{n}f_{ki}d_{ki}}{\sum_{t=1}^{T}\sum_{i=1}^{m}\sum_{k=1}^{n}f_{ki}}\;$$

$$S.t.\;f_{ki}\geq 0$$

$$\overset{m}{\underset{i=1}{\sum }}f_{ki}\leq c_{qk},\;1\leq k\leq n$$

$$\overset{n}{\underset{k=1}{\sum }}f_{ki}\leq c_{pi\;},1\leq i\leq m$$

$$\overset{n}{\underset{k=1}{\sum }}\overset{m}{\underset{i=1}{\sum }}f_{ki}=min\left(\overset{m}{\underset{i=1}{\sum }}c_{pi}\;,\;\overset{n}{\underset{k=1}{\sum }}c_{qk}\;\right)$$

where $f_{ki}$ is the consumption of conversion from $w_{qk}$ to $w_{pi}$ and $d_{ki}$ indicates the distance between $w_{qk}$ and $w_{pi}$, as calculated by:

(7) $$d_{ki}=∥v\left(w_{qk}\right)-v\left(w_{pi}\right){∥}_{2}\;$$

and then the checking-in behavior similarity $cs_{k,i}$ can be calculated as follows:

(8) $$cs_{k,i}=\left(1-EMD\left(u_{k},u_{i}\right)\right)\;$$

Accordingly, we can gain the similarity between $u_{k}$ and $u_{i}$ by:

(9) $$s_{k,i}=gs_{k,i}\cdot cs_{k,i}\;$$

and further, the preference of the targeted user $u_{i}$ influenced by trust users can be estimated as follows:

(10) $$U_{i,j}=\frac{\sum_{u_{k}\in TC}TR_{k,i}\cdot s_{k,i}\cdot C_{k,j}}{\sum_{u_{k}\in TC}TR_{k,i}\cdot s_{k,i}}\;$$

### 3.4. Trust Location-Based Collaborative Filtering

We measure the correlation between locations from two aspects. One is the geographical correlation. Considering that the POIs in $Niche_{i}$ is personalized, we develop an adaptive bandwidth-based kernel density estimation for the personalized geographical correlation. $u_{i}$ has visited POI set $Niche_{i}=\{l_{1},l_{2},\ldots ,l_{q},$ $\ldots ,l_{n}\}$. $r_{i,q}$ is the frequency of $u_{i}$ checking in at $l_{q}$, which is the weight of $l_{q}$. The larger $r_{i,q}$ is, the more $u_{i}$ prefers $l_{q}$. Based on $Niche_{i}$, the geographical correlation can be calculated as follows:

(11) $$P^{g}\left(l_{j}|Niche_{i}\right)=\frac{1}{N}\left(\overset{n}{\underset{q=1}{\sum }}r_{i,q}\cdot K_{Hh_{i}}\left(l_{j}-l_{q}\right)\right)\;$$

and

(12) $$N=\overset{n}{\underset{q=1}{\sum }}r_{i,q}\;\;$$

where $l_{j}$ is visited by $u_{k}\in TC,\;l_{j}\in Niche_{k}$, thereby enhancing the trust of $l_{j}$. $K_{Hh_{i}}\left(l_{j}-l_{q}\right)$ is a kernel function with respect to the adaptive bandwidth $h_{i}$ based on the fixed bandwidth H, including two global bandwidths $\left(H_{1},H_{2}\right)$ which are calculated according to ${\left(4\sigma^{5}/3n\right)}^{1/5}=1.06\sigma n^{-1/5}$ [40], where σ is the standard deviation of the geographical coordinates of $l_{q}$ and $l_{p}$ ($l_{q},l_{p}\in Niche_{i}$):

(13) $$H_{1}=1.06n^{-\frac{1}{5}}\sqrt{\frac{1}{N}{\left(\overset{n}{\underset{q=1}{\sum }}r_{i,q}x_{q}-\frac{1}{N}\overset{n}{\underset{p=1}{\sum }}r_{i,p}x_{p}\right)}^{2}}\;$$

and

(14) $$H_{2}=1.06n^{-\frac{1}{5}}\sqrt{\frac{1}{N}{\left(\overset{n}{\underset{q=1}{\sum }}r_{i,q}y_{q}-\frac{1}{N}\overset{n}{\underset{p=1}{\sum }}r_{i,p}y_{p}\right)}^{2}}\;$$

We further propose to obtain $h_{i}$ for $Niche_{i}$ by utilizing the fixed bandwidth H, as follows:

(15) $$h_{i}={\left(\hat{P}\left(l_{q}|\;L_{i}\right)\cdot Z^{-1}\right)}^{-\beta }\;$$

(16) $$\hat{P}\left(l_{q}|\;L_{i}\right)=\frac{1}{N}\left(\overset{n}{\underset{p=1}{\sum }}r_{i,p}\cdot K_{H}\left(l_{q}-l_{p}\right)\right)\;$$

and

(17) $$K_{H}\left(l_{q}-l_{p}\right)=\frac{1}{2\pi H_{1}H_{2}}\exp\left(-\frac{\left(x_{q}-x_{p}\right)}{2H_{1}^{2}}-\frac{\left(y_{q}-y_{p}\right)}{2H_{2}^{2}}\right)\;$$

where $K_{H}\left(l_{q}-l_{p}\right)$ is a standard kernel function of H consisting of $\left(H_{1}-H_{2}\right)$. β is a sensitive parameter, 0≤β≤1, and the larger β is, the more sensitive $h_{i}$ is to $\hat{P}\left(l_{q}|\;L_{i}\right)$. Z represents the geometric mean of $\hat{P}\left(l_{q}|\;L_{i}\right)$, calculated as follows:

(18) $$Z=\sqrt[{n}]{\prod_{q=1}^{n}\hat{P}(l_{q}|L_{i})}$$

Then, $K_{Hh_{i}}(l_{j}-l_{q})$ can be calculated as follows:

(19) $$K_{Hh_{i}}(l_{j}-l_{q})=\frac{1}{2\pi H_{1}H_{2}h_{i}^{2}}exp(-\frac{\left(x_{j}-x_{q}\right)}{2H_{1}h_{i}^{2}}-\frac{\left(y_{j}-y_{q}\right)}{2H_{2}h_{i}^{2}})$$

where $\left(x_{q},y_{q}\right)$ and $\left(x_{q},y_{q}\right)$ correspond to the geographical coordinates of $l_{q}$ and $l_{j}$, respectively.

Next is the textual content correlation. The textual content of a POI illustrates the profile and equipment which shows what the user prefers. Through exploring the correlations between visited POIs and non-visited POIs, we can capture the potential POIs for users. We employ a topic model to capture contents and measure the correlation between them. Currently, latent Dirichlet allocation (LDA) is one of the most popular techniques for drawing topics. However, it fails to consider the temporal influence on the topic. We employ a beta distribution model to normalize the time from 0 to 1 in a day based upon the consideration that time is intrinsically continuous rather than discrete [18,19,41]. Essentially, we draw POIs’ contents by topics and place textual contents of the same POIs together; thus, we can obtain a larger document set for each POI, which is represented as a random mixture of potential topics. Afterward, we will draw the topic features from documents using the word distribution with timestamps. The detailed steps are as follows:

Draw a multinomial $\Phi_{z}$ from a Dirichlet prior β for each topic *z*: $\Phi_{z}|\beta ∼\mathrm{Dirichlet}(\beta )$.

Draw a multinomial $\theta_{d}$ from a Dirichlet prior α for each document *d* for the location $l_{j}$: $\theta_{d}|\alpha ∼\mathrm{Dirichlet}(\alpha )$.

Draw a topic $z_{di}$ from multinomial $\theta_{d}$ for each word $w_{di}$ in document *d* for the location $l_{j}$: $z_{di}|\theta_{d}∼\mathrm{Multinomial}(\theta_{d})$.

Draw a word $w_{di}$ from multinomial $\Phi_{z_{di}}$ for each word $w_{di}$ in document *d* for the location $l_{j}$: $w_{di}|\Phi_{z_{di}}∼\mathrm{Multinomial}(\Phi_{z_{di}})$.

Draw a timestamp $\Gamma_{di}$ from Beta $\Psi_{z_{di}}$ for each word $w_{di}$ in document *d* for the location $l_{j}$: ${𝒯}_{di}|\Psi_{z_{di}}∼Beta(\Psi_{z_{di}})$.

We propose to employ Gibbs sampling to achieve the topic draw and obtain the conditional distribution based on the chain rule:

(20) $$P(Z_{di}|w,𝒯,Z_{-di},\alpha ,\beta ,\Psi )=(m_{dz_{di}}+\alpha_{z_{di}}-1\times \frac{n_{z_{di}w_{di}}+\beta_{w_{di}}-1}{\sum_{v=1}^{V}(n_{z_{di}v}+\beta_{v})-1}\times \frac{{\left(1-{𝒯}_{di}\right)}^{\Psi_{z_{di}1^{-1}}}{{𝒯}_{di}}^{\Psi_{z_{di}2^{-1}}}}{Beta(\Psi_{z_{di}1},\Psi_{z_{di}2}}$$

where $z_{-di}$ indicates the topic assignments for all tokens $w_{di}$ in document *d* for location $l_{q}$. $𝒯∽Beta(\Psi_{z_{di}1},\Psi_{z_{di}2})$ is updated as follows:

(21) $$\Psi_{z_{di}1}\leftarrow {𝒯}_{z}(\frac{{𝒯}_{z}(1-{𝒯}_{z})}{s_{z}^{2}}-1)$$

and

(22) $$\Psi_{z_{di}2}\leftarrow (1-{𝒯}_{z})(\frac{{𝒯}_{z}(1-{𝒯}_{z})}{s_{z}^{2}}-1)$$

where ${𝒯}_{z}$ and $s_{z}^{2}$ indicate the timestamp sampling mean and covariance to topic z in profiles, respectively.

To summarize, the textual content correlation can be calculated as follows:

(23) $$P^{c}(l_{j}|Niche_{i})=\prod_{l_{q}\in Niche_{i},i\in d}P(Z_{di}|w,\Gamma ,Z_{-di},\alpha ,\beta ,\Psi )$$

Accordingly, we can gain the correlation between $l_{j}$ and $Niche_{i}$, and the preference of the targeted user $u_{i}$ influenced by trust locations can be estimated as follows:

(24) $${ℒ}_{i,j}=TR_{k,i}\cdot P^{g}(l_{j}\in Niche_{k}|Niche_{i})\cdot P^{c}(l_{j}\in Niche_{k}|Niche_{i})$$

### 3.5. Fused Model

We integrate $U_{i,j}$ and ${ℒ}_{i,j}$ with the product rule, which has been widely applied to the fusing of different context factors, showing high robustness [42,43,44]:

(25) $${ℋ}_{i,j}=U_{i,j}\cdot {ℒ}_{i,j}$$

Indeed, the context factor plays an important role in predicting users’ POIs, especially in data sparsity and cold-start problems. Meanwhile, user preference is much more important. In general, matrix factorization-based user preference modeling is common. Let $p_{i}$ be *K*-dimensional potential vector for $u_{i}$, $p_{i}\in P$,and $q_{j}$ be *K*-dimensional potential vector for $l_{j}$, $q_{j}\in Q$. Then, user preference can be denoted as $I_{i,j}=p_{i}^{T}q_{j}$. Accordingly, fused user preference models can be simply divided into two kinds. One is the Gaussian-distribution-based probability model, which weights non-zero entities as 1 and missing entities (zero entities) as 0. Another is the weighted matrix factorization model (WMF) [45], which weighs all entities as non-zero values in terms of check-in frequency. The details are as follows:

(26) $$W_{i,j}=1+\log\left(1+10^{\varepsilon }\cdot r_{i,j}\right)\;$$

where $W_{i,j}$ is the user preference weight, and ε is an adjustable parameter used to control the growth rate of $W_{i,j}$ with increasing $r_{i,j}$. Taking the significance of missing entities into account, we adopt WMF as the basic fused framework for POI recommendation, as follows:

(27) $$\underset{U,L}{\min}G=\overset{\left|U\right|}{\underset{i=1}{\sum }}\overset{\left|L\right|}{\underset{j=1}{\sum }}W_{i,j}{\left(C_{i,j}-\left(I_{i,j}+{ℋ}_{i,j}\right)\right)}^{2}+\lambda_{p}\overset{\left|U\right|}{\underset{i=1}{\sum }}{\left|\left|p_{i}\right|\right|}^{2}+\lambda_{q}\overset{\left|L\right|}{\underset{j=1}{\sum }}{\left|\left|q_{i}\right|\right|}^{2}\;$$

where $\lambda_{p}$ and $\lambda_{q}$ are the regularization coefficients. Note that $I_{i,j}$ is still quite sparse in Equation (27). Therefore, we introduce the POI category factor into it. For example, if $u_{i}$ repeatedly visits $l_{j}$ that is a wetland park, $u_{i}$ may prefer this kind of POI. Consequently, the POI category factor can help model user preference and alleviate data sparsity. Let $v_{c_{j}}$ be the *K*-dimensional potential vector that represents the category c for $l_{j}$, c∈C, and $v_{c}\in V$. We can thus improve user preference $I_{i,j}$ as:

(28) $$I_{i,j}=p_{i}^{T}\left(q_{j}+\gamma \cdot v_{c_{j}}\right)\;$$

where γ is a trade-off parameter used to balance POI category influence to $I_{i,j}$. Accordingly, we rewrite G as follows:

(29) $$\underset{U,L}{\min}G=\overset{\left|U\right|}{\underset{i=1}{\sum }}\overset{\left|L\right|}{\underset{j=1}{\sum }}W_{i,j}{\left(C_{i,j}-\left(I_{i,j}+{ℋ}_{i,j}\right)\right)}^{2}+\lambda_{p}\overset{\left|U\right|}{\underset{i=1}{\sum }}{\left|\left|p_{i}\right|\right|}^{2}+\lambda_{q}\overset{\left|L\right|}{\underset{j=1}{\sum }}{\left|\left|q_{j}\right|\right|}^{2}+\lambda_{v}\overset{\left|C\right|}{\underset{c=1}{\sum }}{\left|\left|v_{c}\right|\right|}^{2}\;$$

where we introduce a regularization term $\sum_{c=1}^{\left|C\right|}{\left|\left|v_{c}\right|\right|}^{2}$ for the POI category, and $\lambda_{v}$ is the corresponding regularization coefficient. Furthermore, we observe that if $u_{i}$ has visited $l_{j}$ ($C_{i,j}=1$), then $I_{i,j}+{ℋ}_{i,j}$ will fit to $C_{i,j}=1$. When ${ℋ}_{i,j}$ is smaller, $I_{i,j}$ may be larger to make up for $I_{i,j}+{ℋ}_{i,j}\approx 1$. For instance, $l_{j}$ is more distant from $u_{i}$, but $u_{i}$ may prefer $l_{j}$. Conversely, when $I_{i,j}$ is smaller, ${ℋ}_{i,j}$ may be larger to make up for $I_{i,j}+{ℋ}_{i,j}\approx 1$. For instance, $u_{i}$ has a weak interest in $l_{j}$, but $l_{j}$ is more nearby to $u_{i}$. Therefore, the context factor is essential when predicting POIs. While if $C_{i,j}=0$, $I_{i,j}+{ℋ}_{i,j}$ will fit to $C_{i,j}=0$, thus $I_{i,j}<0$ due to ${ℋ}_{i,j}>0$, which is unreasonable. The reason is that $C_{i,j}=0$ does not mean that $u_{i}$ is not interested in $l_{j}$, and maybe $u_{i}$ does not know it exists. Surely, we can not conclude that $u_{i}$ is interested in $l_{j}$, so that we believe that the better method is only $I_{i,j}$ is used to fit the user check-in. Finally, we present the refined objective function G as follows:

(30) $$\underset{U,L}{\min}G=\overset{\left|U\right|}{\underset{i=1}{\sum }}\overset{\left|L\right|}{\underset{j=1\left(u_{i},l_{j}\in R\right)}{\sum }}W_{i,j}{\left(C_{i,j}-\left(I_{i,j}+{ℋ}_{i,j}\right)\right)}^{2}+\overset{\left|U\right|}{\underset{i=1}{\sum }}\overset{\left|L\right|}{\underset{j=1\left(u_{i},l_{j}\notin R\right)}{\sum }}I_{i,j}^{2}\;+\lambda_{p}\overset{\left|U\right|}{\underset{i=1}{\sum }}{\left|\left|p_{i}\right|\right|}^{2}+\lambda_{q}\overset{\left|L\right|}{\underset{j=1}{\sum }}{\left|\left|q_{j}\right|\right|}^{2}+\lambda_{v}\overset{\left|C\right|}{\underset{c=1}{\sum }}{\left|\left|v_{c}\right|\right|}^{2}\;$$

To optimize G, we need to update matrix P, Q, and V. Considering that the user-POI rating matrix R is sparse where most missing entities exist, we will mainly deal with missing entities in the update process, such as $\sum_{u_{i},l_{j}\notin R}I_{i,j}^{2}$. Notably, there is no context factor in $\sum_{u_{i},l_{j}\notin R}I_{i,j}^{2}$, thus we can preprocess it offline to reduce time consumption. We develop an optimization method similar to eALS [46], which updates an element at a time instead of a vector. The advantage of the optimization is that it can avoid a large amount of time consumption caused by matrix transpose. Let $x_{i,f}$, $y_{j,f}$, and $z_{c,f}$ be the elements for P, Q, and V, respectively, and the following is achieved:

(31) $$x_{i,f}=\frac{\sum_{j\in R_{i}}{𝒲}_{i,j}h_{j,f}\left(C_{i,j}-\left(I_{i,j}^{x}+{ℋ}_{i,j}\right)\right)-\sum_{j\notin R_{i}}h_{j,f}I_{i,j}^{x}}{\sum_{j\in R_{i}}{𝒲}_{i,j}h_{j,f}^{2}+\sum_{j\notin R_{i}}h_{j,f}^{2}+\lambda_{p}}\;=\frac{\sum_{j\in R_{i}}h_{j,f}\left({𝒲}_{i,j}\left(C_{i,j}-{ℋ}_{i,j}\right)-I_{i,j}^{x}\left({𝒲}_{i,j}-1\right)\right)}{\sum_{j\in R_{i}}\left({𝒲}_{i,j}-1\right)h_{j,f}^{2}+\psi_{f,f}^{h}+\lambda_{p}}-\frac{-\sum_{e\neq f}x_{i,e}\psi_{f,e}^{h}}{\sum_{j\in R_{i}}\left({𝒲}_{i,j}-1\right)h_{j,f}^{2}+\psi_{f,f}^{h}+\lambda_{p}}\;\;\;$$

where $h_{j,f}$ and $I_{i,j}^{x}$ are indicated as follows:

(32) $$h_{j,f}=y_{j,f}+\gamma v_{c_{j},f}\;$$

and

(33) $$I_{i,j}^{x}=I_{i,j}-x_{i,f}h_{j,f}\;$$

and $\psi_{f,e}^{h}$ is an element for the defined cache matrix $\Phi^{h}$:

(34) $$\Phi^{h}=\overset{\left|L\right|}{\underset{j=1}{\sum }}\left(q_{j}+\gamma v_{c_{j}}\right){\left(q_{j}+\gamma v_{c_{j}}\right)}^{T}\;$$

Similarly,

(35) $$y_{j,f}=\frac{\sum_{i\in R_{j}}x_{i,f}\left(W_{i,j}\left(C_{i,j}-{ℋ}_{i,j}\right)-I_{i,j}^{y}\left(W_{i,j}-1\right)\right)}{\sum_{i\in R_{j}}\left(W_{i,j}-1\right)x_{i,f}^{2}+\psi_{f,f}^{p}+\lambda_{q}}-\frac{\sum_{e\neq f}y_{j,e}\phi_{f,e}^{p}+\gamma \sum_{e=1}^{K}v_{c_{j},f}\psi_{f,e}^{p}}{\sum_{i\in R_{j}}\left(W_{i,j}-1\right)x_{i,f}^{2}+\psi_{f,f}^{p}+\lambda_{q}}\;$$

and

(36) $$z_{c,f}=\frac{\sum_{j\in {𝒞}_{c}}\left(\sum_{i\in R_{j}}\gamma x_{i,f}(W_{i,j}\left(C_{i,j}-{ℋ}_{i,j}\right)-I_{i,j}^{z}\left(W_{i,j}-1\right))\right)}{\sum_{j\in {𝒞}_{c}}\left(\sum_{j\in R_{i}}\gamma^{2}({𝒲}_{i,j}-1\right)x_{i,f}^{2}+\gamma^{2}\psi_{f,f}^{p})+\lambda_{v}}-\frac{\sum_{j\in {𝒞}_{c}}(\gamma^{2}\sum_{e\neq f}z_{c_{j},f}\psi_{f,e}^{p}+\gamma \sum_{e=1}^{K}y_{j,e}\psi_{f,e}^{p})}{\sum_{j\in {𝒞}_{c}}(\sum_{j\in R_{i}}\gamma^{2}\left(W_{i,j}-1\right)x_{i,f}^{2}+\gamma^{2}\psi_{f,f}^{p})+\lambda_{v}}\;$$

where $\psi_{f,e}^{p}$ is an element for the defined cache matrix $\Phi^{p}=P^{T}P$. $I_{i,j}^{y}$ and $I_{i,j}^{z}$ indicate:

(37) $$I_{i,j}^{y}=I_{i,j}-x_{i,f}y_{j,f}\;$$

and

(38) $$I_{i,j}^{z}=I_{i,j}-\gamma x_{i,f}z_{c_{j},f}\;$$

### 3.6. Time Complexity Analysis

The main time consumption of BiTCF is in the process of updating $x_{i,f}$, $y_{j,f}$, and $z_{c,f}$. We take Equation (31) as an example, where most of the time consumption comes from missing items $\sum_{j\notin R_{i}}h_{j,f}I_{i,j}^{x}$ and $\sum_{j\notin R_{i}}h_{j,f}^{2}$, and thus we are the first to deal with the missing items. According to $\sum_{j\notin R_{i}}h_{j,f}I_{i,j}^{x}=$ $\sum_{k\neq f}x_{i,k}\sum_{j=1}h_{j,f}h_{j,k}-\sum_{j\in R_{i}}h_{j,f}I_{i,j}^{x}$, we can obtain the item that is not related to users of $\sum_{j=1}h_{j,f}h_{j,k}$ (similarly,$\;\sum_{j\notin R_{i}}h_{j,f}^{2}$). Therefore, $\Phi^{h}$ (Equation (34)) can be updated offline. Except for the offline part, the online time complexity of calculating P is $O\left(\left|U\right|K^{2}+\left|R\right|K\right)$. Correspondingly, the time complexity of calculating both Q and V is $O\left(\left|L\right|K^{2}+\left|R\right|K\right)$ in Equations (35) and (36), respectively. Therefore, the time complexity of our proposed model is $O\left(\left(\left|U\right|+\left|L\right|\right)K^{2}+\left|R\right|K\right)$, which is the optimal time complexity among state-of-the-art models based on matrix factorization frameworks, to the best of our knowledge.

## 4. Experiments

### 4.1. Datasets

We adopt Gowalla and Foursquare datasets, which are widely used LBSN datasets. Following the preprocessing performed by a previous work [47], we remove the users who have less than 10 check-in records or have checked in less than 5 POIs and POIs that have been visited by less than 10 users. Afterward, we gain the datasets as shown in Table 2. The Gowalla dataset contains 301,191 check-in records where there are 4159 users, 24,919 POIs, 225 categories, and a 0.291% density. The Foursquare dataset contains 289,467 check-in records, where there are 3475 users, 21,657 POIs, 157 categories, and a 0.385% density. To evaluate the performance of our proposed model, we randomly select 80% check-in records from each user as the training set and the remaining 20% as the testing set. In the training and test processing, our proposed model takes about 200 epochs and 250 epochs to converge steadily on Gowalla and Foursquare for each run, which is analyzed in Section 4.5.

### 4.2. Baselines

- Context-influence-enhanced models
  (1) **ASMF** [17]: ASMF is a fused weighted matrix factorization with social factor which defines three types of friendships, including social friends, location friends, and neighboring friends, for POI recommendation.
  (2) **TA** [18]: TA is a temporal factor-enhanced collaborative filtering model that recommends POIs to a given user at a specific time.
  (3) **ST-RNet** [19]: ST-RNet is a spatiotemporal recommender network model which learns the cross-features and the combined features of users, POIs, and time together based on neural network.
  (4) **SSTPMF** [8]: SSTPMF is a POI recommendation model integrating social spatiotemporal information into probabilistic matrix factorization, which develops a multivariable inference approach using the latent social space, geographical space, and POI category space similarities for POI recommendation.

- Trust-enhanced models
  (1) **SPTW** [12]: SPTW is a social pertinent trust walker model for POI recommendation, which is modeled by calculating the level of trust between users in social networks. Combining high probability location category algorithm, SPTW can generate POI recommendation lists.
  (2) **TECF** [15]: TECF is a trust-enhanced collaborative filtering model, which fuses the geographic factor, temporal factor, and trust relationship learned by DeepWalk model running over the user covisiting network, for POI recommendation.

**BiTCF**: Our proposed model.

### 4.3. Parameter Settings

We tune the parameters by employing the grid search used in [6,8,17] and set different parameter values for the baselines on two datasets to achieve their best performance. In BiTCF, we set the required parameters as $\theta ,b,\;\alpha ,\beta ,\varepsilon ,\gamma ,\;\lambda_{p},\;\lambda_{q}$, and $\lambda_{v}$. We tune the parameters by employing grid search to confirm the optimal settings on two datasets, including θ in {0, 0.1, 0.2, 0.3, 0.4, 0.45, 0.5, 0.55, 0.6, 0.7, 0.8, 0.9, 1},  b in {10, 15, 20, 25, 30, 35, 40, 45, 50, 55,60},  α in {0.1, 0.2, 0.3, 0.4, 0.5, 0.6, 0.7, 0.8, 0.9},  β in {0.1, 0.2, 0.3, 0.4, 0.5, 0.6, 0.7, 0.8, 0.9, 1},  ε in {1, 2, 3, 4, 5},  γ in {0, 0.2, 0.4, 0.6, 0.8, 1}, as well as $\lambda_{p},\;\lambda_{q}$, and $\lambda_{v}$ in {0.001, 0.01, 0.1, 1, 10, 100, 1000}. Finally, on Gowalla, the optimal settings are θ=0.6, b=45, α=0.7, β=0.5, ε=3, γ=0.4, $\lambda_{p}=\lambda_{q}=0.01$, and $\lambda_{v}=200$. On Foursquare, the optimal settings are θ=0.55, b=30, α=0.8, β=0.5, ε=2, γ=0.6, $\lambda_{p}=\lambda_{q}=0.01$, and $\lambda_{v}=100$.

### 4.4. Evaluation Metrics

We adopt two widely used metrics to evaluate the performance of BiTCF and the other baseline models, such as precision@k and recall@k with k={5, 10, 20, 40}, as follows:

(39) $$precision@k=\frac{1}{\left|U\right|}\overset{\left|U\right|}{\underset{i=1}{\sum }}\frac{\left|S_{i}\left(k\right)\cap^{\;}Test_{i}\right|}{k}\;$$

and

(40) $$recall@k=\frac{1}{\left|U\right|}\overset{\left|U\right|}{\underset{i=1}{\sum }}\frac{\left|S_{i}\left(k\right)\cap^{\;}Test_{i}\right|}{\left|Test_{i}\right|}\;$$

where $S_{i}\left(k\right)$ indicates the top-*k* POI list recommended to $u_{i}$, and $Test_{i}$ is a POI set consisting of the POIs in the testing set visited by $u_{i}$. All of the baseline models run over the test data 10 times, and each metric is also calculated 10 times and averaged to decrease errors.

### 4.5. Experimental Results

Firstly, we evaluate the performance of all models with top-5 recommendation results in different dimensions, K={5, 10, 20, 40}. The results are shown in Table 3. When K<10, the fused collaborative filtering models with context factors, i.e., TA and TECF, and the deep-learning-based model ST-RNet have more advantages over the other models based on matrix factorization. In particular, TECF is the most outstanding because it considers the trust relationship between users. When K≥10, the matrix factorization-based recommendation models, i.e., ASMF, SSTPMF, SPTW, and our proposed BiTCF, show excellent performance, among which SSTPMF and BiTCF performs best. When K=20, BiTCF outperforms the other 6 models. Compared with SSTPMF, without considering the trust factor and TECF with the trust relationship between users, BiTCF considers the trust filtering from the views of users and locations. On the Gowalla dataset, the improvements over SSTPMF and TECF are 12.9% and 11.8%, respectively, as well as 14.5% and 13.4%, respectively, on the Foursquare dataset. Let K=20 in other experiments we implement.

Furthermore, we evaluate the performance of all models with top-*k* recommendation results in the dimension K=20, k={5, 10, 20, 40}. The experimental results are shown in Table 4. Evidently, with the increase in *k*, each model shows a trend of decreasing precision@k and increasing recall@k. The reason is that more POIs recommended to users contain more user preferences, while the POIs which may be visited will reduce. Specifically, the models integrating fewer context factors, i.e., ASMF and TA, are inferior to the models fusing more context factors, i.e., ST-RNet, SSTPMF, and BiTCF. Among the trust-enhanced models, TECF outperforms SPTW, and both are inferior to BiTCF. This is because BiTCF performs more sufficient trust filtering from the views of users and locations using various factors.

In addition, considering that the cold-start problem (given-*n*) is a serious challenge for recommender systems, we evaluate the performance of all models when each user only has visited the given *n* POIs, where n={3, 5, 10}. The experimental results are shown in Table 5. As we can see, with the increase in n, each model shows a trend of increasing precision@5 and recall@5 because users visited more POIs, thus helping the models learn user preferences. Furthermore, the context factor plays an important role in predicting user preferences. The models integrating fewer context factors, i.e., ASMF, TA, and SPTW, are inferior to the models fusing more context factors, i.e., ST-RNet, SSTPMF, TECF, and BiTCF. Because BiTCF integrates user similarity, geographical factor, temporal factor, textual content factor, POI category factor, and trust factor, which can effectively and efficiently work in the cold-start problem, it is the best-performing model.

**Figure 2:**
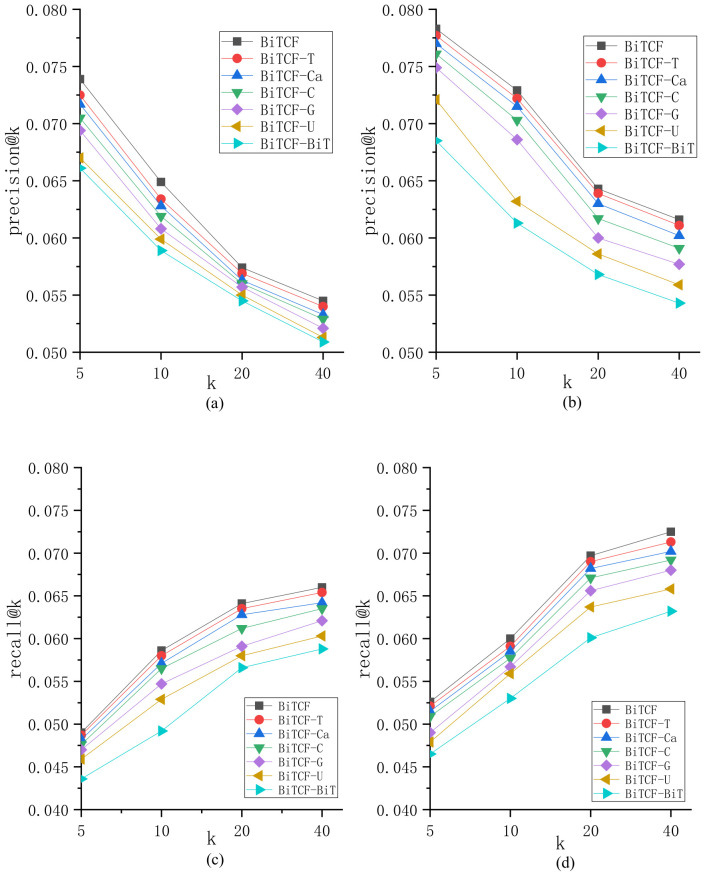
Comparison of different factor contributions: (**a**,**c**) on Gowalla; (**b**,**d**) on Foursquare.

We propose BiTCF for personalized POI recommendation via fusing 6 kinds of context factors, including user similarity, geographical factor, temporal factor, textual content factor, POI category factor, and trust factor. We evaluate the contributions of different factors to explore their roles. We name the model eliminating user similarity as BiTCF-U, the model eliminating geographical factor as BiTCF-G, the model eliminating temporal factor as BiTCF-T, the model eliminating textual content factor as BiTCF-C, the model eliminating POI category factor as BiTCF-Ca, and the model eliminating trust factor as BiTCF-BiT. The experimental results are shown in Figure 2. BiTCF has the best performance over all models, demonstrating the effectiveness of fusing these factors. The effectiveness of these factors is different: trust factor > user similarity > geographical factor > textual content > POI category > temporal factor.

**Figure 3:**
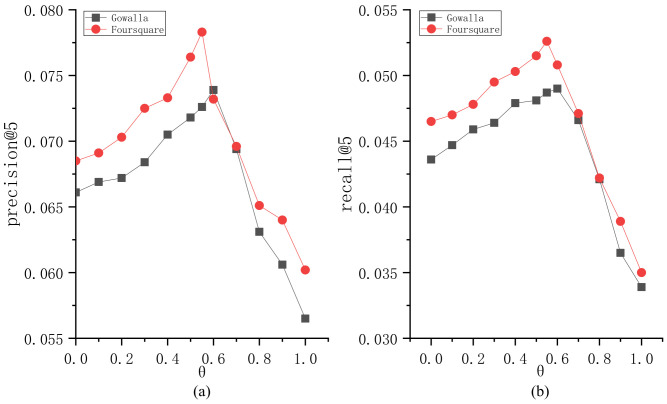
The sensitivity analysis of θ. (**a**) precision@5; (**b**) recall@5.

**Figure 4:**
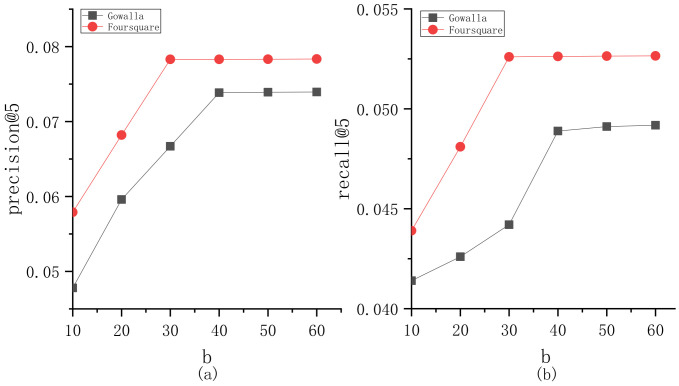
The sensitivity analysis of b. (**a**) precision@5; (**b**) recall@5.

**Figure 5:**
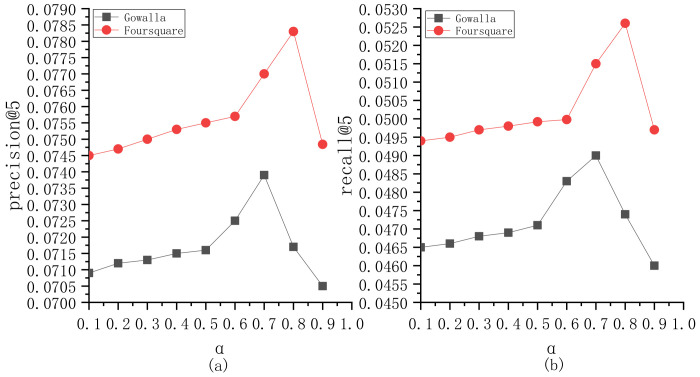
The sensitivity analysis of α. (**a**) precision@5; (**b**) recall@5.

**Figure 6:**
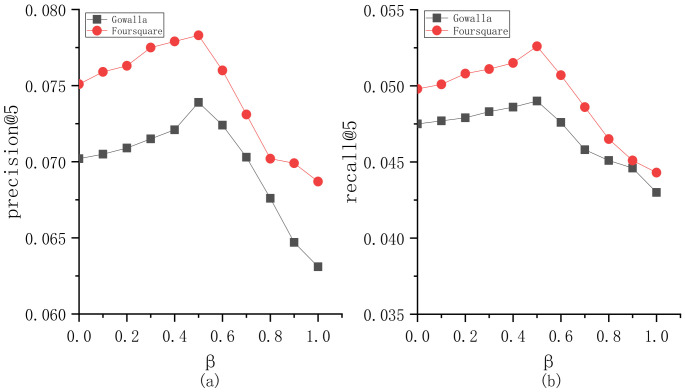
The sensitivity analysis of β. (**a**) precision@5; (**b**) recall@5.

**Figure 7:**
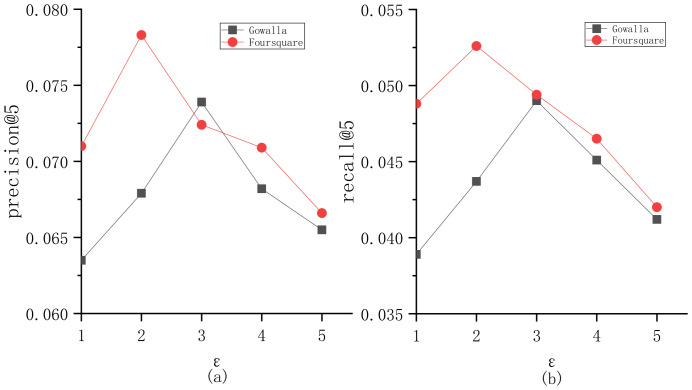
The sensitivity analysis of ε. (**a**) precision@5; (**b**) recall@5.

**Figure 8:**
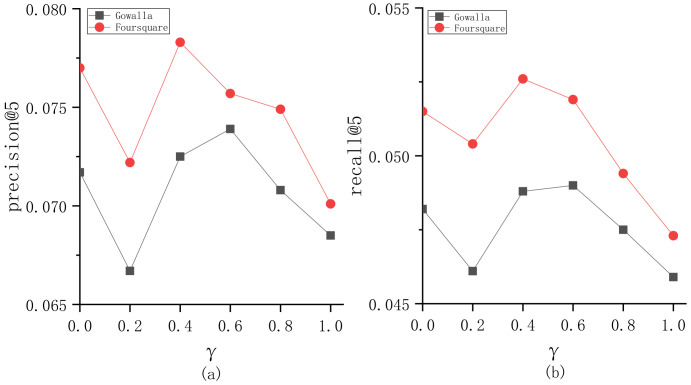
The sensitivity analysis of γ. (**a**) precision@5; (**b**) recall@5.

To explore the parameter sensitivity, we implement the experiments to understand the roles of different parameters in BiTCF. Here, there are 6 important parameters, i.e., θ,b,  α,  β,  ε,  γ. We still employ the same test set mentioned in Section 4.1 and select precision@5 and recall@5 as the metrics. Furthermore, we adapt the control variates for parameters to implement the experiments, namely we vary a parameter value while keeping the others constant. Figure 3 shows the sensitivity analysis of θ. On Gowalla, θ=0.6, resulting in an optimal performance of BiTCF, and on Foursquare, θ=0.55. When θ<0.6 and 0.55 on Gowalla and Foursquare, respectively, the noise data are not adequately filtered, leading to limited efficiency. While θ>0.6 and 0.55, the trust filtering is excessive, which seriously influences the number of available trustworthy users, resulting in the limited fitting of context factors. Figure 4 shows the sensitivity analysis of b. With the increase in b, BiTCF has a better performance, and the performance achieves an approximate optimal near b=45 (Gowalla) and b=30 (Foursquare). The larger b causes more neighbor users to participate in calculation, among which include more users who are distant from the target user (b>45 and 30), causing a vainly increased calculation consumption. Figure 5 illustrates the sensitivity analysis of α. α balances the importance between *DTR* and *IDTR*. On Gowalla, when α=0.7, BiTCF achieves the optimal performance, and α=0.8 on Foursquare. Figure 6 shows the sensitivity analysis of β. When β=0, the kernel density estimation method with adaptive bandwidth is reduced to the method with fixed bandwidth. BiTCF performs best when β=0.5. If β<0.5, $P^{g}\left(l_{j}|Niche_{i}\right)$ has low sensitivity to user check-in records, and if β>0.5, $P^{g}\left(l_{j}|Niche_{i}\right)$ has a much higher sensitivity to user check-in records, causing fitting over. Figure 7 shows the sensitivity analysis of ε. Near ε=3 and ε=2 on Gowalla and Foursquare, respectively, BiTCF achieves the best performance. The reason is that our proposed model is based on a WMF framework, which assigns a non-zero weight to each missing entity and a weight calculated by using ε to the non-zero entities. If ε is smaller, there is little difference between the non-zero entities and missing entities, which impacts the role of non-zero entities. While if ε is bigger, the role of the missing entities is impacted. Figure 8 illustrates the sensitivity analysis of γ. γ trades off the impact of the POI category factor on BiTCF. As we can see, different γ values have a great impact on the performance of the model, and the model achieves the optimal performance when γ=0.6 on Gowalla and γ=0.4 on Foursquare.

**Figure 9:**
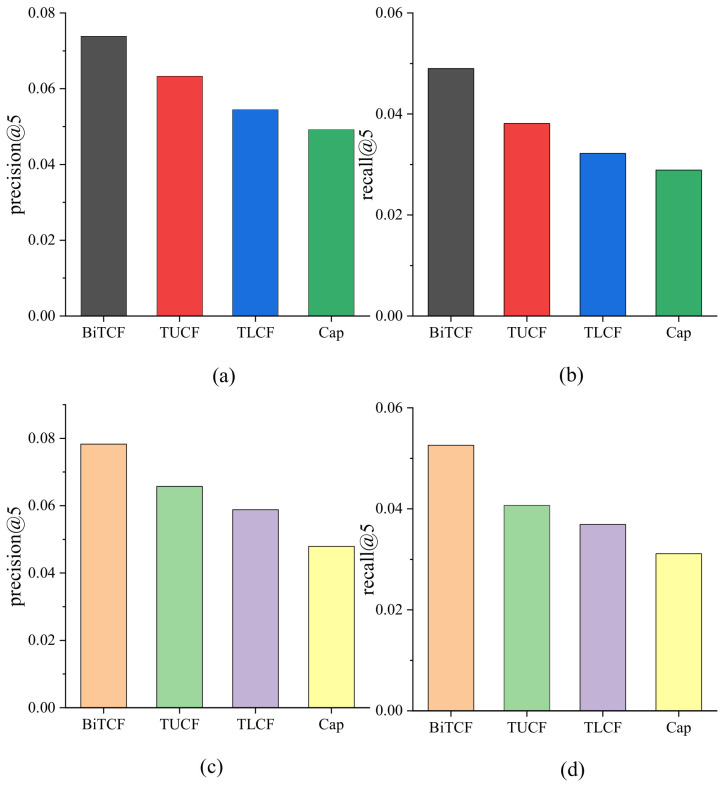
Comparison of the effectiveness of four modules: (**a**,**b**) on Gowalla; (**c**,**d**) on Foursquare.

BiTCF is combined with trust-user-based collaborative filtering (TUCF), trust-location-based collaborative filtering (TLCF), and POI category-enhanced historical preference (CaP) modules. To evaluate the effectiveness of the above three modules, we implement the ablation experiment and the results are shown Figure 9. TUCF achieves a better performance compared with TLCF and CaP, and TLCF is superior to CaP.

**Figure 10:**
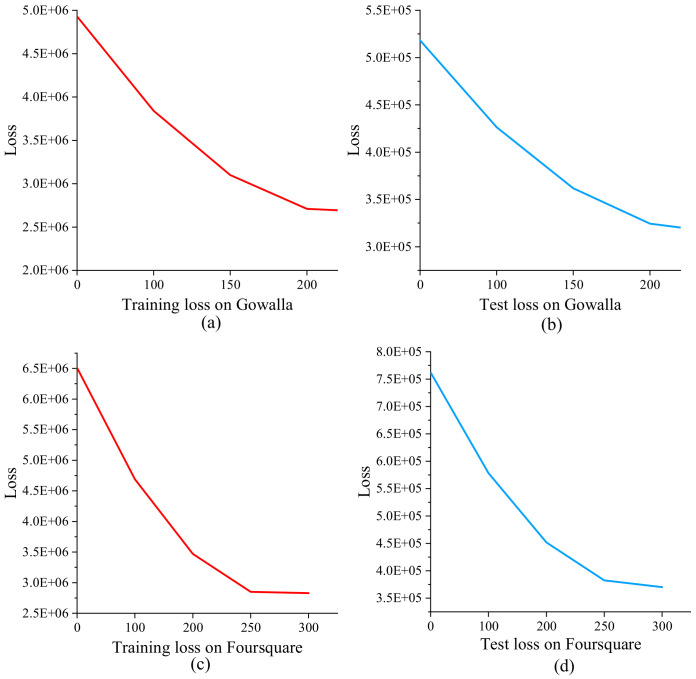
Training and test loss of BiTCF@: (**a**,**b**) on Gowalla; (**c**,**d**) on Foursquare.

To evaluate the practical computing time complexity in training and test processing, we provide the converge speed of BiTCF on Gowalla and Foursquare datasets, as shown in Figure 10. BiTCF takes about 200 epochs and 250 epochs to converge steadily on Gowalla and Foursquare, which indicates the low computing time complexity of both training and test processing.

## 5. Conclusions

To address the trustworthiness and data sparsity problems in POI recommender systems, we propose a novel POI recommendation model with bidirectional trust-enhanced collaborative filtering. Firstly, we model a trustworthy community based on trust relationships. Secondly, we perform the trust filtering from the views of users and locations. From the views of users, taking the influence of temporal factors on user similarity into account, we propose a time-aware similarity measurement fusing trust factor. From the view of locations, considering the roles of geographical and textual content factors, we propose a location correlation measurement with geographical, textual content, and trust factors. Thirdly, we integrate the POI category factor into a weighted matrix factorization to learn user preference. To summarize, we have developed a fusion with two kinds of integrating methods for the trust-enhanced collaborative filtering and the user preference models.

Compared with the state-of-the-art models, the proposed model further considers the location trust alongside the user trust, and it refines context influence models for fitting the user preference more precisely. In addition, the model develops a novel fusion strategy to perform fusion between the user preference model and the trust-enhanced context models. Consequently, the model could even better facilitate user life, e.g., the user $u_{i}$ visits a series of POIs in a period of time, and the model then recommends some unvisited POIs to him/her based on historical records after comprehensively considering spatiotemporal factors, which can help users to accelerate retrieval and filter information from massive data.

We implement experiments on two widely used LBSN datasets to evaluate our proposed model. The model is deployed on a server with configuration (CPU: AMD Ryzen9 5950X 16-Core Processor 3.40 GHz; RAM: 128 G (32 GB DDR4-3600 DDR4 × 4); GPU: NVIDIA GeForce RTX3090 (24 GB) × 2; ROM: 1T SSD + 4T HDD) and run by Python + MongoDB. The experimental results demonstrate that our proposed model is effective and efficient and outperforms the state-of-the-art trust-enhanced POI recommendation models. In the future, we will focus on ensuing POI recommendations, with an emphasis on the check-in sequence and the corresponding dependence relations between different POIs in the sequence of user real-time demands.

## Figures and Tables

**Table 1:** The descriptions of key notions.

Notions	Descriptions
*U*, *L*, C	The user set, POI set, POI category set
*R*	The user-POI rating matrix
$r_{i,j}$	The frequency or rating of $u_{i}$ on $l_{j}$, $r_{i,j}\in R$
$\left[TR_{k,i}\right]$	The trust weighted matrix
$DTR_{k,i}$	The direct trust relationship between $u_{i}$ and $u_{k}$
$IDTR_{k,i}$	The indirect trust relationship between $u_{i}$ and $u_{k}$
$\left[s_{k,i}\right]$	The user similarity matrix
$gs_{k,i}$	The geographical similarity between $u_{i}$ and $u_{k}$
$cs_{k,i}$	The check-in behavior similarity between $u_{i}$ and $u_{k}$
$\left[U_{i,j}\right]$	The trust user influence matrix
$\left[{ℒ}_{i,j}\right]$	The trust location influence matrix
$P^{g}\left(l_{j}|Niche_{i}\right)$	The geographical correlation between $l_{j}$ and $Niche_{i}$
$P^{c}\left(l_{j}|Niche_{i}\right)$	The textual content correlation between $l_{j}$ and $Niche_{i}$
$\left[{ℋ}_{i,j}\right]$	The trust-enhanced context factor fused matrix
$\left[I_{i,j}\right]$	The user preference matrix
P	The *K*-dimensional potential matrix for users
Q	The *K*-dimensional potential matrix for POIs
V	The *K*-dimensional potential matrix for POI categories

**Table 2:** Statistics of the dataset.

Dataset	|Users|	|POIs|	|Categories|	|Check-Ins|	Density%
Gowalla	4159	24919	225	301191	0.291
Foursquare	3475	21657	157	289467	0.385

**Table 3:** Comparisons on the performance of the models with top-5 POIs in different dimensions.

Dataset	*K*	Metrics	ASMF	TA	ST-RNet	SSTPMF	SPTW	TECF	BiTCF
Gowalla	5	*precision*@5	0.0512	0.0571	0.0620	0.0522	0.0508	**0.0649**	0.0566
		*recall*@5	0.0341	0.0353	0.0382	0.0343	0.0336	**0.0444**	0.0347
	10	*precision*@5	0.0574	0.0571	0.0620	0.0624	0.0565	**0.0649**	0.0644
		*recall*@5	0.0359	0.0353	0.0382	0.0401	0.0348	**0.0444**	0.0419
	20	*precision*@5	0.0617	0.0571	0.0620	0.0657	0.0605	0.0649	**0.0739**
		*recall*@5	0.0362	0.0353	0.0382	0.0433	0.0349	0.0444	**0.0490**
	40	*precision*@5	0.0613	0.0571	0.0620	0.0651	0.0607	0.0649	**0.0731**
		*recall*@5	0.0371	0.0353	0.0382	0.0437	0.0360	0.0444	**0.0475**
Foursquare	5	*precision*@5	0.0553	0.0609	0.0635	0.0568	0.0550	**0.0673**	0.0598
		*recall*@5	0.0361	0.0355	0.0399	0.0366	0.0356	**0.0472**	0.0389
	10	*precision*@5	0.0597	0.0609	0.0635	0.0645	0.0589	0.0673	**0.0684**
		*recall*@5	0.0374	0.0355	0.0399	0.0429	0.0366	**0.0472**	0.0468
	20	*precision*@5	0.0621	0.0609	0.0635	0.0682	0.0613	0.0673	**0.0783**
		*recall*@5	0.0371	0.0355	0.0399	0.0460	0.0362	0.0472	**0.0526**
	40	*precision*@5	0.0607	0.0609	0.0635	0.0684	0.0603	0.0673	**0.0755**
		*recall*@5	0.0364	0.0355	0.0399	0.0455	0.0355	0.0472	**0.0492**

**Table 4:** Comparisons on the performance of the models with top-k POIs in dimension *K*=20.

Dataset	Metrics	ASMF	TA	ST-RNet	SSTPMF	SPTW	TECF	BiTCF
Gowalla	*precision*@5	0.0617	0.0571	0.0620	0.0657	0.0605	0.0649	**0.0739**
	*recall*@5	0.0362	0.0353	0.0382	0.0433	0.0349	0.0444	**0.0490**
	*precision*@10	0.0536	0.0503	0.0555	0.0588	0.0533	0.0568	**0.0649**
	*recall*@10	0.0423	0.0411	0.0447	0.0489	0.0405	0.0505	**0.0586**
	*precision*@20	0.0475	0.0452	0.0502	0.0537	0.0476	0.0520	**0.0574**
	*recall*@20	0.0500	0.0493	0.0519	0.0562	0.0471	0.0577	**0.0641**
	*precision*@40	0.0446	0.0417	0.0468	0.0501	0.0432	0.0492	**0.0545**
	*recall*@40	0.0528	0.0517	0.0540	0.0583	0.0495	0.0600	**0.0660**
Foursquare	*precision*@5	0.0621	0.0609	0.0635	0.0682	0.0613	0.0673	**0.0783**
	*recall*@5	0.0371	0.0355	0.0399	0.0460	0.0362	0.0472	**0.0526**
	*precision*@10	0.0552	0.0535	0.0566	0.0609	0.0541	0.0602	**0.0729**
	*recall*@10	0.0427	0.0419	0.0462	0.0524	0.0420	0.0531	**0.0600**
	*precision*@20	0.0492	0.0472	0.0510	0.0562	0.0483	0.0543	**0.0643**
	*recall*@20	0.0501	0.0490	0.0523	0.0590	0.0472	0.0608	**0.0697**
	*precision*@40	0.0461	0.0441	0.0473	0.0535	0.0440	0.0522	**0.0616**
	*recall*@40	0.0524	0.0509	0.0541	0.0622	0.0486	0.0635	**0.0725**

**Table 5:** Comparisons on the performance of the models in the cold-start problem.

Dataset	*n*	Metrics	ASMF	TA	ST-RNet	SSTPMF	SPTW	TECF	BiTCF
Gowalla	3	*precision*@5	0.0166	0.0150	0.0184	0.0201	0.0158	0.0199	**0.0227**
		*recall*@5	0.0145	0.0141	0.0159	0.0176	0.0138	0.0179	**0.0208**
	5	*precision*@5	0.0179	0.0157	0.0197	0.0218	0.0168	0.0214	**0.0263**
		*recall*@5	0.0162	0.0145	0.0181	0.0200	0.0142	0.0150	**0.0227**
	10	*precision*@5	0.0192	0.0169	0.0210	0.0238	0.0179	0.0225	**0.0300**
		*recall*@5	0.0176	0.0152	0.0195	0.0213	0.0150	0.0218	**0.0251**
Foursquare	3	*precision*@5	0.0174	0.0155	0.0188	0.0207	0.0162	0.0202	**0.0234**
		*recall*@5	0.0149	0.0142	0.0165	0.0184	0.0141	0.0189	**0.0210**
	5	*precision*@5	0.0189	0.0172	0.0205	0.0229	0.0182	0.0226	**0.0274**
		*recall*@5	0.0156	0.0155	0.0180	0.0210	0.0150	0.0216	**0.0233**
	10	*precision*@5	0.0207	0.0188	0.0220	0.0243	0.0200	0.0239	**0.0311**
		*recall*@5	0.0185	0.0176	0.0203	0.0225	0.0174	0.0232	**0.0260**

## Data Availability

The data used to support this study are available from the corresponding author upon request.
